# Predictive factors and clinical effect of optimized cardiac resynchronization therapy

**DOI:** 10.3892/etm.2012.802

**Published:** 2012-06-11

**Authors:** GUO-JUN XU, TIAN-YI GAN, BAO-PENG TANG, YI-TONG MA, YU ZHANG, JIN-XIN LI, YAN-YI ZHANG, JIANG WANG, QI TANG, CHUN-MEI WANG, YAO-DONG LI, JIANG-HUA ZHANG

**Affiliations:** Department of Cardiology, First Affiliated Hospital, Xinjiang Medical University, Urumqi 830054, P.R. China

**Keywords:** heart failure, cardiac resynchronization therapy, echocardiography, prediction of response, follow-up

## Abstract

The aim of this study was to assess the effectiveness of cardiac resynchronization therapy (CRT) by intracardiac delay optimization using echocardiography. Sixty-five patients were implanted with a CRT device randomly assigned to receive simultaneous biventricular pacing or echo-optimized sequential CRT. Forty-two patients were defined as responders and 23 patients were classified as non-responders. During a 12-month follow-up period, the positive response rate, QRS duration, New York Heart Association class, mitral insufficiency grade, left ventricular end-systolic volume and LV end-diastolic volume were similar in the optimized and non-optimized groups (P>0.05), whereas 6-minute walking distance, quality-of-life score, left ventricular (LV) ejection fraction and aortic velocity time integral were significantly improved in the optimized group (P<0.05). The baseline QRS durations of the responders and non-responders were similar (P>0.05), whereas heart failure aetiology, clinical and echocardiographic measurements showed significant differences (P<0.05). The mean decrease in QRS duration after 12 months of CRT used for separating responders and non-responders was significantly different (P<0.05), and significant differences were observed in the mean decrease of QRS duration between responders and non-responders (P<0.05). Echocardiographic optimization may further improve the effectiveness of CRT. Moreover, severe mitral regurgitation and greater LV volume are likely to indicate a poor response to CRT.

## Introduction

Despite improvements in pharmacologic treatment, many patients with heart failure have severe and persistent symptoms and their prognosis remains poor (1,2). Such patients commonly have periods of delayed myocardial activation and contraction, leading to cardiac dyssynchrony. In a series of trials lasting up to six months, cardiac resynchronization therapy (CRT) decreased symptoms and improved exercise capacity, quality of life and ventricular function (3–5). Moreover, a decrease in rehospitalization for heart failure and improved long-term survival compared with optimal medical therapy has been demonstrated (4,6). Current CRT devices allow manipulation of the atrioventricular (AV) and interventricular (VV) timings in order to maximize left ventricular (LV) filling and stroke volume. Intracardiac delay optimization of biventricular pacing devices has become an important tool to improve CRT therapy and the quality of life of non-responders (7,8). However, multiple single center and multicenter trials have provided controversial data on the beneficial effects of AV and VV interval optimization on cardiac performance and clinical status (9–11). Therefore, the purpose of this study was to assess whether or not systematic intracardiac delay optimization using echocardiography is superior to a fixed nominal AV and VV delay as demonstrated by improved ventricular function and LV geometry during long-term follow-up.

Despite current selection criteria, up to 40% of patients treated with CRT do not benefit (12,13). It has been suggested that QRS duration may not be the optimal criterion when selecting patients for CRT (14,15). Identifying reliable predictors of the effectiveness of CRT remains a major challenge in clinical practice, particularly from the perspective of patient selection. Accordingly, in the present study, we performed an analysis to identify baseline predictive factors of a positive response to CRT.

## Materials and methods

### Patients

In this prospective study, our center analyzed 65 patients, 46 patients implanted with CRT-P (biventricular pacemakers) and 19 patients implanted with CRT-D (biventricular cardioverter-defibrillators), from January 2003 to December 2008. Patients were selected according to current guidelines for CRT (16,17): i) severe heart failure [New York Heart Association (NYHA) class III or IV], ii) depressed left ventricular ejection fraction (LVEF; ≤35%), iii) QRS showing a left bundle branch block configuration with a duration ≥120 msec, iv) normal sinus rhythm and v) optimized medical therapy. Patients with right bundle branch block, nonspecific intraventricular conduction delay, ventricular pacing or atrial fibrillation were excluded (16–18). Patients who had experienced a major cardiovascular event in the previous six weeks, those who had conventional indications for a pacemaker or an implantable defibrillator and those with heart failure requiring continuous intravenous therapy were excluded (17,18). Also excluded were patients with atrial arrhythmias, since such patients do not benefit from the atrial component of resynchronization. In order to obtain unbiased data regarding cardiac improvement, standard and individually optimized heart failure medication [including β blockers, angiotensin-converting enzyme (ACE) inhibitors or angiotensin (AT)-1 receptor blockers at the maximally tolerated dose and spironolactone at 25 mg/day] remained unchanged 3 months prior to implantation of CRT in all patients.

### Study protocol

All patients underwent a clinical examination and echocardiographic evaluation prior to CRT and after long-term follow-up. Evaluation of clinical status included assessment of NYHA class and patient’s quality-of-life score. The quality-of-life score was assessed using the Minnesota Living with Heart Failure Questionnaire, which contains 21 questions concerning a patient’s perception of the effects of heart failure on daily life activities (19). Questions were scored from 0–5, resulting in a total score of 0–105, with the highest score reflecting the worst quality of life (19). In addition, exercise capacity was evaluated by assessing 6-minute walking distance. QRS duration was measured using a surface electrocardiograph (ECG) before and after implantation of the CRT device. ECGs were recorded at a speed of 25 mm/sec and evaluated by two independent observers without knowledge of the patients’ clinical status. QRS duration was measured using the widest QRS complex in leads II, V1 and V6. The 65 CRT recipients were randomly assigned at a 1:1 ratio to receive simultaneous biventricular pacing (the non-optimized group) or optimized sequential CRT (the optimized group).

Patients with an improvement of ≥1 grade in NYHA class were considered clinical responders to CRT (20,21). Patients with a decrease ≥10% in left ventricular end-systolic volume (LVESV) at 12-month follow-up were considered echocardiographic responders to CRT (22). Clinical and echocardiographic data were analyzed by two independent observers blinded to all other patient data. In addition, patients who succumbed to progressive heart failure before the 12-month follow-up assessment were classified as non-responders. The study protocol was approved by the local Ethics Committee and informed consent to participate in the investigation was obtained from each patient prior to enrolment (23).

### Implantation of a biventricular pacemaker

During pacemaker implantation, the LV pacing lead was inserted transvenously via the subclavian route (24). After a coronary sinus venogram was obtained, the LV pacing lead (Attain LV lead, Medtronic, Minneapolis, MN, USA) was inserted through the coronary sinus with the help of an 8Fr guiding catheter and positioned in the venous system, preferably in the (postero-) lateral vein. Thereafter, the right atrial and ventricular leads were positioned. The CRT device and lead implantation was successful in all patients without major complications (device models 8042, 7277, 7279, Medtronic). All 65 implanted devices were combined CRT-P or CRT-D devices with programmable AV and VV intervals. Effective biventricular pacing (defined as effective pacing and proper sensing on both ventricular leads and proper function of the atrial lead in patients with sinus rhythm) and ventricular pacing percentage should not decrease below 80% according to device data (2,3,24).

### Echocardiographic AV and VV delay optimization method

All echocardiographic data were analyzed offline by the same investigator, blind to all programming information. For each parameter, three consecutive cardiac cycles were analyzed and the average value was taken.

The ultrasound system used was a Vivid 7 (GE Medical Systems, Milwaukee, WI, USA). The LVEF, LVESV and LV end-diastolic volumes (LVEDV) were calculated according to the biplane modified Simpson’s rule (25). The degree of mitral regurgitation (MR) was assessed according to the American Society of Echocardiography guidelines in orthogonal apical echocardiographic images as the average of the maximal areas of the Doppler colour flow-mapped regurgitant jet within the left atrium and also as the ratio of the regurgitant jet area to the left atrial area (26). Pulsed-Doppler velocity signals of transmitral flow were recorded at 100 mm/sec with the sample volume at the tips of the mitral valve leaflets. The aortic velocity time integral (VTIa) was measured by Doppler analysis of the transaortic flow. Peak velocities were measured during rapid LV filling (E-wave) and atrial contraction (A-wave) and the velocity ratio (E/A) was calculated.

This study protocol was based on echo-guided optimization of AV and VV delay performed within 48 h of implantation and then repeated during the follow-up for patients in the optimized group. The heart rate was stable (±5 bpm). AV delay optimization was performed during simultaneous biventricular pacing. AV delays were analyzed between 60 and 200 msec, in steps of 10 msec. Utilizing pulsed Doppler analysis of the transmitral flow, the programmed AV delay that provided the longest LV filling time and EA interval without interruption of the A-wave was chosen. VV delay optimization was performed following AV delay programming. We analyzed VV intervals ranging from −80 msec (LV pacing first) to +80 msec (right ventricular pacing first), in steps of 10 msec. VV delay was optimized by measuring VTIa and optimal VV delay was determined by maximizing VTIa.

### Statistical analysis

The data were analyzed using the statistical software program SPSS V.12.0.1. Summary data are expressed as mean ± standard deviation (SD) or percentage of patients. The comparison of the baseline characteristics between the groups was performed with the independent sample t-test and the χ^2^ test for categorical variables. Continuous variables within and between groups were compared using two-sided paired and unpaired Student’s t-test. Categorical data were compared using the Cochran-Armitage test for trends. A two-tailed P<0.05 was considered to indicate a statistically significant difference.

## Results

Table I shows the baseline characteristics of the entire study population. The study population included 65 patients; a nonischemic dilated cardiomyopathy was present in 65% of patients and ischaemic cardiomyopathy in 35%. During the entire study period, 4 of 65 patients (6%) succumbed to progressive heart failure. The implantation of a CRT-P or CRT-D was associated with a significant reduction in the risk of mortality, as has been identified in previous studies (27–30). The current study revealed that the same patients in different stages had various non-constant AV and VV interphases. The best optimizing AV interphase is 100–140 msec and the best optimizing VV interphase is 10–30 msec. The NYHA class, 6-minute walking distance and quality-of-life score were significantly improved in the overall study population at 12 months (all P<0.01). Furthermore, LVEF and VTIa were significantly greater; LVESV and LVEDV were significantly lower; the grade of mitral insufficiency was significantly reduced and the mean QRS duration was significantly shorter following CRT device implantation at 12-month follow-up (all P<0.05; Table I).

No significant differences in the baseline variables between the optimized and non-optimized groups were observed (Table II). Two patients succumbed in each group. Analyses were conducted to assess the behavior of clinical, electrocardiographic and echocardiographic variables in the two groups, by comparing them in the periods before and after the surgery (7 days, 6 months and 1 year after). The two groups presented a similar mean decrease in QRS duration, mitral insufficiency grade, NYHA class, LVESV and LVEDV; however, there was no significant difference between the two groups (all P>0.05). The 6-minute walking distance, quality-of-life score, LVEF and VTIa were significantly improved in the optimized group (all P<0.05; Table II).

As shown in Table III, 42 patients were defined as responders, while 23 patients were classified as non-responders. The baseline QRS durations of the responders and non-responders were similar (P>0.05). No significant differences in baseline results for age, gender or duration of symptoms between the two groups were observed (all P>0.05), whereas the baseline results for NYHA class, quality-of-life score, 6-minute walking distance, mitral insufficiency grade, heart failure etiology, LVEF, LVESV and LVEDV revealed significant differences between the two groups (all P<0.05, Table III).

During the 12-month follow-up, the functional capacity and echocardiography measurements demonstrated significant improvement in the responder group (all P<0.05) and the mean decreases in QRS duration for the responder and non-responder groups were significantly different (all P<0.05; Table III).

As shown in Table IV, the response positive rate exhibited a higher tendency in the optimized group but was not significantly improved compared with that in the non-optimized group (P>0.05).

## Discussion

Our results indicate that the use of biventricular stimulation to resynchronize LV contraction may improve clinical outcomes in patients with a prolonged QRS interval and advanced symptomatic heart failure as a result of moderate to severe LV systolic dysfunction. Our results add to those of earlier, short-term studies that evaluated the effects of CRT on exercise tolerance, symptoms of heart failure and the quality of life (2–4,6,29). Taken together, these data indicate that, in a population with advanced heart failure and an increased QRS interval, CRT improves the majority of factors that affect the quality of life. This study suggests that the synchronous LV contraction pattern, provided by CRT, is a major determinant of LV function improvements. Moreover, our data demonstrates that CRT-P or CRT-D significantly increases the survival benefit, resulting in a 6% mortality rate at 12-month follow-up, which is comparable with the findings of previous studies (27–30). This reduced mortality from progressive heart failure may be related to the favorable effects of the devices used to treat the clinical syndrome of heart failure, which is consistent with a favorable effect of CRT on systolic function.

In summary, in our selected patients, CRT-P and CRT-D improved the clinical course of chronic heart failure due to a dilated cardiomyopathy. The pacemaker is associated with reduced symptoms, improved exercise tolerance and quality of life and reduced mortality.

Several studies have reported acute hemodynamic improvements following post-implant AV delay optimization and tailored biventricular pacing (31). Furthermore, CRT optimization may provide a more homogeneous ventricular activation pattern, in terms of prolongation of LV filling time and reduction in interventricular and intraventricular dyssynchrony (6,7). Sequential biventricular pacing with the VV delay optimized, enhances the response to CRT compared to simultaneous CRT, as it improves systolic function and reduces MR and LV volumes in patients with heart failure and electromechanical delay (8,9). VV delay optimization has been shown to improve NYHA class and LVEF at follow-up (10). Moreover, a recent study reported the effects on myocardial performance index (MPI) of echocardiographic optimizations performed at implantation and after 6 months. The optimal AV and VV delay combinations were shown to change at the 6-month evaluation point, which implied a significant hemodynamic improvement, in terms of MPI, that was still maintained at 12 months (6,10,11).

In accordance with previous observations (6,31), we observed a beneficial effect of CRT on systolic function and LV volumes. This is of interest as simultaneous biventricular pacing improves cardiac performance compared with the native rhythm and hemodynamics may be further improved by individually programming AV and VV delay. However, as suboptimal pacemaker programming post-CRT may be a determinant for lack of optimal benefit, optimization of AV and VV delay in addition to CRT may lead to a further increase of myocardial function (7–9). The progressive decrease in LV volumes recorded during the 12-month follow-up confirms the dynamic characteristics of CRT-induced LV reverse remodeling. Even if the reprogramming of a CRT device is only one of the multiple factors interacting in the combined therapy of heart failure patients, it is possible that changes in optimal AV and VV programming during 12-month follow-up may further improve the beneficial effect of CRT on LV geometry and function.

Temporal variations of echo-guided optimized AV and VV delays during follow-up have been reported previously (11). Additionally, there is a significant progressive lengthening of the LV diastolic filling time and transmitral velocity during early diastolic filling (10). This is of interest as these parameters may reflect improvements in diastolic function (10,11). Echocardiography allows simultaneous assessment of LV systolic and diastolic function as well as evaluation of temporal inter- and intra-ventricular events, valve regurgitation and particularly MR and pulmonary arterial pressure (9). This clearly demonstrates, in the echo-optimization, that the differences in delay occurring in our patients may significantly limit the benefit of CRT by means of suboptimal delay settings. This study revealed that the same patients in different stages have various AV and VV interphases, which are not constant.

The rationale of CRT is to correct cardiac dyssynchrony through biventricular pacing. As a consequence, the presence of a wide QRS complex (≥120 msec) is included in the selection criteria for CRT (16–18).

We studied the value of QRS duration prior to implantation of a CRT device in 65 patients to evaluate the level of response. In addition, we evaluated the decrease in QRS duration after 12 months of CRT to predict the response to CRT. The present study aimed to evaluate the predictive value of QRS duration for clinical and echocardiographic responses to CRT in a selected study population. No significant differences were observed in QRS duration at baseline between the responders and non-responders; however, significant differences were observed in the mean decrease of QRS duration between the two groups at 12-month follow-up. A correlation was observed between the change of baseline QRS duration and improvement in exercise capacity, functional status, quality-of-life score, LVEF and LV volume at 12-month follow-up. Moreover, our study demonstrated that, besides the change of QRS duration, ischemic heart disease, impaired LV function, severe MR and greater LV volume were predictors for non-response; patients with those characteristics were likely to have a less satisfactory response to CRT. Accordingly, it has been suggested that the duration of the QRS complex at baseline may not be the optimal criterion for selection of patients for CRT.

This is a small pilot study performed in a single center. Due to the small number of patients, no multivariate analysis of the several parameters and no calculation of predictive values could be performed. We used formerly popular parameters that were relatively easy and not time-consuming to examine; however, measurement of these clinical parameters is subjective and imprecise with low reproducibility and high inter- and intra-observer variability, as is known from the PROSPECT study (13).

The modified Simpson’s rule was used for LV volume calculation, which may not be optimal for the spherical shape and asymmetrical contraction in the hearts examined. With the discoordinated contraction in left bundle branch block (LBBB), the smallest cavity area may not truly represent the end of systole, as late activated areas may still be contracting. The choice of echocardiographic parameters should include parameters of three-dimensional echo as well as parameters of radial and circumferential asynchrony. This was not included since the software and equipment was not available for all patients.

Currently, a clear definition of response to CRT is lacking. Clinical and echocardiographic end points are interchangeably used to determine the response to CRT. In the present study, clinical and echocardiographic variables were used to define the response to CRT. Importantly, there is a discrepancy between clinical and echocardiographic responses to CRT. However, despite the growing emphasis on several criteria for the response to CRT, large prospective studies are required to determine which parameter is best for selecting patients for CRT.

## Figures and Tables

**Table I:** Baseline and 12 month clinical data in the study population.

Variable	Baseline	12 month	P-value
Male/female (n)	51/14	49/12	P>0.05
Age (years)	66±9	65±9	P>0.05
Heart failure etiology (n)			
Ischemic cardiomyopathy	23	19	P>0.05
Non-ischemic cardiomyopathy	42	42	P>0.05
Duration of symptoms (years)	7±5	7±5	P>0.05
Mortality (n)	0	4	P>0.05
QRS duration (ms)	181±28	154±36	P<0.01
Mitral insufficiency grade 1/2/3 (n)	25/29/11	36/18/7	P<0.01
NYHA class I/II/III/IV(n)	0/0/41/24	7/35/12/7	P<0.01
Distance walked in 6 min (m)	289±102	356±105	P<0.01
Quality-of-life score	42±21	27±12	P<0.05
LVEF (%)	25±9	31±9	P<0.05
LVESV (ml)	166±68	147±72	P<0.05
LVEDV (ml)	208±74	183±78	P<0.05
VTIa (cm)	12±4	22±6	P<0.05

Unless specified otherwise, values are mean ± standard deviation. NYHA, New York Heart Association; LVEF, left ventricular ejection fraction; LVESV, left ventricular end-systolic volume; LVEDV, left ventricular end-diastolic volume; VTIa, aortic velocity time integral.

**Table II:** Comparison of patient characteristics of the optimized and non-optimized groups at baseline and during follow-up.

Variable	Optimized group (n=31)	Non-optimized group (n=30)	P-value
Age (years)	65±9	67±8	P>0.05
Male/female (n)	25/6	24/6	P>0.05
Heart failure etiology (n)			
Ischemic cardiomyopathy	10	9	P>0.05
Non-ischemic cardiomyopathy	21	21	P>0.05
Duration of symptoms (years)	7±4	7±6	P>0.05
Mitral insufficiency grade 1/2/3 (n)			
Baseline	13/12/6	12/13/5	P>0.05
Follow-up (1 week)	14/14/3	14/13/3	P>0.05
Follow-up (6 months)	18/10/3	17/9/4	P>0.05
Follow-up (12 months)	19/9/3	17/9/4	P>0.05
QRS duration (ms)			
Baseline	179±30	182±27	P>0.05
Follow-up (1 week)	153±24	155±21	P>0.05
Follow-up (6 months)	151±25	156±22	P>0.05
Follow-up (12 months)	151±23	156±24	P>0.05
Quality-of-life score			
Baseline	42±24	42±27	P>0.05
Follow-up (1 week)	32±9	35±12	P>0.05
Follow-up (6 months)	24±10	32±11	P<0.01
Follow-up (12 months)	24±12	32±9	P<0.01
Distance walked in 6 min (m)			
Baseline	287±103	293±105	P>0.05
Follow-up (1 week)	324±89	321±91	P>0.05
Follow-up (6 months)	367±92	334±89	P<0.05
Follow-up (12 months)	368±94	334±89	P<0.05
NYHA class I/II/III/IV(n)			
Baseline	0/0/20/11	0/0/20/10	P>0.05
Follow-up (1 week)	0/20/7/4	0/17/8/5	P>0.05
Follow-up (6 months)	3/21/5/2	2/19/6/3	P>0.05
Follow-up (12 months)	3/20/5/3	2/18/7/3	P>0.05
LVEF (%)			
Baseline	25±8	25±9	P>0.05
Follow-up (1 week)	30±7	29±6	P>0.05
Follow-up (6 months)	34±8	29±8	P<0.05
Follow-up (12 months)	34±9	29±7	P<0.05
VTIa (cm)			
Baseline	12±4	12±4	P>0.05
Follow-up (1 week)	20±5	19±6	P>0.05
Follow-up (6 months)	25±4	20±5	P<0.05
Follow-up (12 months)	25±5	20±4	P<0.05
LVESV (ml)			
Baseline	165±67	166±69	P>0.05
Follow-up (1 week)	156±62	158±70	P>0.05
Follow-up (6 months)	146±71	149±73	P>0.05
Follow-up (12 months)	145±69	149±74	P>0.05
LVEDV (ml)			
Baseline	208±76	209±72	P>0.05
Follow-up (1 week)	191±65	193±74	P>0.05
Follow-up (6 months)	183±75	186±76	P>0.05
Follow-up (12 months)	181±78	185±77	P>0.05

Unless specified otherwise, values are mean ± standard deviation. NYHA, New York Heart Association; LVEF, left ventricular ejection fraction; VTIa, aortic velocity time integral; LVESV, left vetricular end-systolic volume; LVEDV, left ventricular end-diastolic volume.

**Table III:** Comparison of patient characteristics of clinical responders vs. clinical non-responders at baseline and during follow-up.

Variable	Responders (n=42)	Non-responders (n=23)	P-value
Age (years)	65±9	67±8	P>0.05
Male/female (n)	33/9	16/5	P>0.05
Heart failure etiology (n)			
Ischemic cardiomyopathy	8	15	P<0.01
Non-ischemic cardiomyopathy	33	8	P<0.01
Duration of symptoms (years)	7±5	8±5	P>0.05
Mortality (n)	0	4	P<0.05
QRS duration (ms)			
Pre-implantation	181±26	184±27	P>0.05
Post-implantation	152±20^a^	165±30^a^	P<0.05
ΔQRS (ms)	29±23	19±35	P<0.05
NYHA class I/II/III/IV(n)			
Baseline	0/0/31/11	0/0/10/13	P<0.05
Follow-up	7/33/2/0	0/0/12/7	P<0.01
Quality-of-life score			
Baseline	36±24	48±27	P<0.05
Follow-up	12±9	42±24	P<0.01
Distance walked in 6 min (m)			
Baseline	318±102	274±109	P<0.01
Follow-up	365±56	278±87	P<0.01
Mitral insufficiency grade 1/2/3 (n)			
Baseline	25/14/3	0/15/8	P<0.05
Follow-up	36/6/0	0/12/7	P<0.01
LVEF (%)			
Baseline	27±8	24±7	P<0.05
Follow-up	32±8	25±6	P<0.01
LVESV (ml)			
Baseline	158±67	172±73	P<0.05
Follow-up	116±52	169±75	P<0.01
LVEDV (ml)			
Baseline	192±72	226±75	P<0.05
Follow-up	163±65	224±74	P<0.01

Unless specified otherwise, values are mean ± standard deviation. ΔQRS = QRS duration before - QRS duration after CRT implantation.

a P<0.05, QRS duration before vs. after CRT implantation. NYHA, New York Heart Association; LVEF, left ventricular ejection fraction; LVESV, left ventricular end-systolic volume; LVEDV, left ventricular end-diastolic volume.

**Table IV:** Comparison of the response rates of the optimized and non-optimized groups during follow-up.

	Responders (n)	Non-responders (n)	Total (n)	Positive rate (%)
Optimized group (n)	23	10	33	69.69
Non-optimized group (n)	19	13	32	59.37
Total (n)	42	23	65	64.61

χ^2^=0.3729, P=0.5414.
